# Analecta

**Published:** 1842-03-12

**Authors:** 


### ANALECTA.

*Prolapsus of the Bladder and Anterior Paries of the Vagina.—Operation for Episcorraphie.* By Robert T. Lightfoot, Esq., Surgeon, Newcastle-on-Tyne.—Margaret Walton, ætat. 19, domestic servant, unmarried, consulted me about the beginning of July, 1841. She states that towards the end of last April, whilst lifting a heavy beadstead, she "heard her back crack," and immediately after experienced a bearing-down sensation in the pelvis, and a dragging pain in the loins. These symptoms, along with a difficulty in voiding the urine and fæces, continued for ten days or a fortnight, when she first observed a small tumour to protrude from the vagina, which gradually increased until it attained the size of an orange. As soon as the tumour appeared externally the pain at stool ceased; but the necessity to void the urine became so frequent and urgent, that on making the slightest exertion, as in going up stairs, contraction of the bladder was induced, and a few drops of urine passed involuntarily. Her general health now became so much impaired, that she was obliged to give up her situation. At first she could reduce the tumour when in the horizontal position, but for the last six weeks she has been unable to do so. The tumour is at present of the size of the fist, protruding from the vagina and hanging between the thighs: the greater portion of it is formed by the bladder and anterior paries of the vagina, for on introducing a catheter (which must be held perpendicular to the axis of the trunk) the point of it may be felt nearly throughout its whole extent in front. Towards the posterior part the neck of the uterus is observed very much elongated; the os uteri is filled by a semitransparent glairy secretion, and its posterior lip is considerably enlarged; the surface of the tumour is dry, cracked, and partially covered with superficial ulcerations, which secrete a muco-purulent fluid; menstruation regular.

After remaining in bed for twenty-four hours the tumour was easily reduced, but, owing to the capacity of the vagina, it immediately fell down again on making the least exertion, or when standing erect. These manipulations always produce an urgent desire to evacuate the bladder. So long as she remains at rest in the horizontal posture, and the tumour is reduced, she can entirely empty the bladder; but on making any exertion it descends, and the urine dribbles away involuntarily. A bandage and the ordinary contrivances were had recourse to, but without the slightest benefit; and as the patient was wishful to undergo any treatment that afforded the chance of a radical cure, rather than remain in her present state, I determined (in consultation with Mr. Frost of this town) to perform the operation for episcorraphie, as recommended by Dr. Fricke, of Hamburg.

Sept. 2. The bowels having been previously evacuated, and the hair removed from the parts, the patient was placed on a table, in the same position as for lithotomy, without, however, tying the hands and feet. The thighs being well separated by two assistants, I took hold of the left labium and transfixed it obliquely about the middle with a narrow bistoury three-quarters of an inch from the edge, and in such a manner as to include more of the skin than of the mucous membrane; the knife was then carried rapidly downwards in the same direction to the raphe, half an inch or so in front of the anus: the superior attachment of this flap was next divided, by carrying the incision upwards as high as on a level with the meatus urinarius. The same was repeated on the opposite side; after which the frenulum, and other parts included within the angle formed by the union of the two incisions in front of the anus, were carefully dissected off. The two surfaces thus formed extended from opposite the urethra to within half an inch of the anus, each being about two inches long, and varying in breadth from an inch posteriorly to half an inch anteriorly. The hæmorrhage was so trifling, as merely to require the torsion of one small vessel: the oozing having ceased, six strong hempen sutures were passed through the entire thickness of the denuded surfaces, and tied moderately firm. The first one was applied a few lines in front of the anus, and the last one immediately below the meatus urinarius.

A gum-elastic catheter was introduced into the bladder, and the knees were bound together, after which the patient was placed in bed on her left side. The catheter was allowed to remain for the two first days, but owing to the large size of the urethra, part of the urine escaped by the side of the instrument, and, coming in contact with the uniting surface, produced a good deal of irritation; it was therefore removed, and during the five or six following days it was merely introduced occasionally. The antiphlogistic regimen was strictly adhered to for four or five days, during which cold water was constantly applied to the parts, and the vagina occasionally washed out and cleared of coagulated blood, by means of cold water injections. An anodyne was given at bed-time, and repeated for three or four nights, to allay irritation and confine the bowels. Two of the sutures were removed on the fourth day, and the other four on the sixth; at the expiration of which, union by the first intention had taken place throughout the whole extent. The bowels were moved on the eighth day, and an occasional dose of aperient medicine was afterwards given.

In the course of three weeks she was allowed to leave her bed, and walk about a little ; and on the 30th of September she was able to attend to her usual housework : she can retain her urine for from five to six hours, and can evacuate it voluntarily, and without the slightest inconvenience. The bond of union is very firm, and appears like an elongated perineum, extending from the anus to within a quarter of an inch of the urethra. The finger can be introduced into the vagina with great ease, the parts being very dilatable.

Nov. 20. Although the patient has been exposed to considerable exercise, there is not the least return of the prolapsus : on desiring her to strain violently, a portion of the corrugated anterior paries of the vagina becomes visible, but has not the slightest tendency to protrude externally, although the passage into the vagina is enlarged from the longitudinal contraction of the cicatrix.

In performing this operation, I think it would be better not to attempt the union of the posterior part of the labia, but to leave an opening into the vagina between the bond of union and the frenulum sufficiently large to allow of the discharge of the vaginal mucus and menstrual secretion. By adopting this method the operation would be much facilitated; the most troublesome part of it, and, at the same time, the most painful to the patient, being the dissection required for the removal of the frenulum and the parts in front of the anus. During the treatment, likewise, coagulated blood, and the secretions from the vagina and wounds, could be more easily removed ; and in case of considerable inflammation occurring after the operation, cold water could be more readily and continuously applied by means of a syringe. By this modification of the operation, the union of the labia would form a kind of bridge, with two communications into the vagina. The extent of this required, would depend very much upon the case: thus, if there was considerable prolapsus of the bladder and anterior paries of the vagina, it would be necessary to carry the union as far as the meatus urinarius, and the posterior opening might be left large enough to admit the finger. If, on the other hand, the tumour was principally formed by the uterus and posterior wall of the vagina, then it would be advisable to leave the posterior opening just sufficiently large to admit an ordinary-sized gum-elastic catheter, and the outer one must be left of considerable extent; care, however, must be taken to make the bond of union of sufficient extent, so as to allow for the subsequent contraction of the cicatrix, otherwise one or other of the openings might become so large as to allow the inverted mucous membrane gradually to be insinuated through it, and any unusual effort would expose the patient to a return of the complaint.—*London Lancet*, Dec. 4, 1841.

---

*Glanders and Farcy in man.* Translated by B. Daniel, from a paper read before Académie des Sciences at Paris, Nov. 15, 1841.—Glanders and farcy are two diseases of a similar nature to which horses are particularly liable : these affections may be either acute or chronic, and may also be converted from one to the other. Horses affected with either of these disorders ought to be equally objects of dread, as rabid animals, because if these affections be not equally as contagious as rabies, yet they are so to such a degree as to command the greatest precautions. Cases of acute glanders and farcy are found to be more contagious than chronic, although the contagious property of these diseases amongst horses has been disputed, from the fact that out of a given number of healthy animals lodged in the same stable with horses affected with these disorders, all have not contracted the disease. This only proves that the power of its contagion is variable, and facts recently observed have proved, in a most convincing manner, the communication of this disease from horse to man. This will also prove *à fortiori* its contagion from horse to horse. During the last four years, medical journals have recounted more than forty cases of acute and chronic glanders and acute farcy having been developed in men who had been in contact with animals affected with those disorders. Thus the ignorance of the contagious property of these evils has cost those persons their lives, and unless the danger be rendered obvious, will still continue to make victims, since medicine has not the power of curing those who may be attacked ; all we can hope, and that is of much importance, is the discovery of means for preventing the development of so cruel a disease. It may be communicated by inoculation or by infection—by inoculation, when the groom or person who attend the horse have a slight sore by which some portion of the matter discharged from the animal may be absorbed ; by infection, when a person without having any wound, contracts the disease solely by habitation with the affected animal.

This abridged history of glanders and farcy, which M. Littré has written in a popular treatise, is now illustrated by the following case, which M. Berard has communicated to the Academy. It is without example, at least up to the present time, of a man affected with acute glanders communicating the malady to the person who approaches him, and from whom he was receiving aid. M. Rocher, medical student at the hospital of Necker, was charged with the dressing of a patient affected, first with chronic farcy, subsequently with acute glanders, under which he died last month (October.) The dressing of the patient occasioned daily contact between him and the pupil; moreover, the latter, too zealous in the pursuit of science, prolonged the time of his relation with the patient in examining all the symptoms of the disease minutely. M. Rocher took also an active part in the examination of the body after death, and during the time that the nasal fossæ were being sawn he maintained the head immovable by placing his hands over the integuments of the temples and face, which were the seat of the gangrenous eruption of the disease. Such, then, are the circumstances under which M. Rocher contracted the malady. Previous to the death of the groom the pupil had already suffered from an attack of diarrhœa and colic, but it was only in the night after the post-mortem examination that the disease really commenced. M. Rocher awoke in the night with shivering, to which succeeded fever and general pains ; the two following days, though he felt much pain in his joints, he yet rose and left his chamber. The third day the pains became much more acute, and fixed themselves in the left thigh, the right shoulder, and the right side of the chest. On the fifth day M. Berard discovered in the thickness of the thigh and shoulder two tumors, having the characteristics peculiar to farcy, and from that time he prognosticated the most unfavourable termination of the malady. During the following days the tumour of the shoulder was absorbed, whilst that of the thigh became soft and fluctuating. Six days after its appearance it was opened, and the liquid discharged ; it consisted of pus mixed with blood. The discharge was collected and given up to M. Leblanc, a distinguished veterinary surgeon, who inoculated a horse with it on the same day. In the mean time a new tumor, preceded by excessive pain, made its appearance on the internal malleolus of the right foot, and in the course of three days it arrived at suppuration. Finally, a fortnight after the commencement of the disease the skin of the nose became inflamed and painful; the following day the inflammation extended to the cheeks, to the eyelids, and as far as the middle of the forehead; gangrenous phlyctænæ and pustules appeared scattered over the red and swollen parts of the face; the latter symptoms were more marked the following day, when a copious bloody liquid was discharged through the nose; numerous pustules then appeared all over the body, and on the ensuing night M. Rocher died, being sixteen days from the commencement of the disease. The horse inoculated by M. Leblanc died on the same night as M. Rocher, after having exhibited all the symptoms of farcy and acute glanders. The examination of the nasal fossæ presented those lesions which particularly characterise the latter affection. The case which has just been related, says the author in concluding, proves the contagious property of glanders from man to man. It was not by inoculation that M. Rocher caught the disease, he had no excoriation on the fingers or hands during the whole of his attendance on the patient; he neither punctured nor cut himeelf during the post-mortem examination, but always washed his hands carefully after touching his patient. The disease therefore, must have been contracted in consequence of a miasmatic infection analogous to that of variola or scarlatina. In point of science this fact presents great interest, but is of more importance as regards the public health: it shows to what dangers those persons are exposed who approach individuals affected with these disorders: it will induce medical men to take and recommend precautions for avoiding the contagion: even here it ought not to rest, it calls for the interference of government. Let us hope that the authorities, in giving more attention to that part of public hygiene which concerns domestic animals, and by causing horses affected with these disorders to be destroyed, may thus put a limit to this horrible disease, which up to the present time has always proved fatal, whoever it has attacked.—*London Loncet, Nov.* 17, 1841.

---

*Parasitic Leeches.*—M. Guyon presented on the 11th of October, some specimens of a species of a minute leach, the hæmopis vorax, to the Academy of Sciences in Paris, which he had collected during his residence in Africa, and which he found on several occasions in the larynx and trachea of man and animals. In an ox, he found twelve of these animals in the mouth and fauces, five upon the epiglottis, four in the larynx, and six in the commencement of the trachea. Twelve hours after the death of the ox they were yet living, and adhered closely to the mucous membrane of the animal, requiring to be touched with alcohol in order to induce them to quit their hold. When applied to rabbits and fowls they took with great avidity. Some of these animals were very minute and filiform; and in this state they enter the air-passages of man. M. Guyon also found them in the horse.—*Lond. Lancet, Dec.* 18, *from Gaz. Med. de Paris.*

---

*The Medical Examiner* is published every Saturday by *J. C. Turnpenny*, N. E. corner of Spruce and Tenth streets. Terms—*three dollars* a year for one copy, or *five dollars* for two copies sent to the same post office, in all cases in advance. Postage the same as on newspapers. Letters to be addressed,—post-paid,—to the Editors of the Medical Examiner, Philadelphia.
*Reynell Coates, M. D.*, Acting Editor.
*J. B. Biddle, M. D.*, and *W. W. Gerhard, M. D.*, Co-editors.
*J. B. Biddle, M. D.*, Proprietor.

